# Review of skin irritation/corrosion Hazards on the basis of human data: A regulatory perspective

**DOI:** 10.2478/v10102-012-0017-2

**Published:** 2012-06

**Authors:** David Basketter, Dagmar Jírova, Helena Kandárová

**Affiliations:** 1 DABMEB Consultancy Ltd, Sharnbrook, Unidet Kingdom; 2 National Institute of Public Health, Prague, Czech Republic; 3 MatTek In Vitro Life Science Laboratories, Bratislava, Slovak Republic

**Keywords:** skin irritation hazard, human 4 h human patch test, regulatory toxicology

## Abstract

Regulatory classification of skin irritation has historically been based on rabbit data, however current toxicology processes are transitioning to *in vitro* alternatives. The *in vitro* assays have to provide sufficient level of sensitivity as well as specificity to be accepted as replacement methods for the existing *in vivo* assays. This is usually achieved by comparing the *in vitro* results to classifications obtained in animals. Significant drawback of this approach is that neither *in vivo* nor *in vitro* methods are calibrated against human hazard data and results obtained in these assays may not correspond to situation in human.

The main objective of this review was to establish an extended database of substances classified according to their human hazard to serve for further development of alternative methods relevant to human health as well as resource for improved regulatory classification. The literature has been reviewed to assemble all the available information on the testing of substances in the human 4 h human patch test, which is the only standardized protocol in humans matching the exposure conditions of the regulatory accepted *in vivo* rabbit skin irritation test.

A total of 81 substances tested according to the defined 4 h human patch test protocol were found and collated into a dataset together with their existing *in vivo* classifications published in the literature. While about 50% of the substances in the database are classified as irritating based on the rabbit skin test, on using the 4 h HPT test, less than 20% were identified as acutely irritant to human skin. Based on the presented data, it can be concluded that the rabbit skin irritation test largely over-predicts human responses for the evaluated chemicals. Correct classification of the acute skin irritation hazard will only be possible if newly developed *in vitro* toxicology methods will be calibrated to produce results relevant to man.

## Introduction

One of the most important advances in regulatory toxicology has been the implementation of the Globally Harmonised System (GHS) for the identification, classification and labelling of substances, mixtures and preparations (United Nations-Economic Commission for Europe, 2009). The hazard associated with a single chemical substance or a mixture of 2 or more substances refers to their intrinsic property to cause a particular effect, in this case, acute skin irritation and corrosion. In regulatory terms, skin corrosion represents irreversible damage to the skin, whereas skin irritation is characterised by clinical evidence of inflammation which is entirely reversible.

In the past, the potential of a substance or preparation to cause skin irritation or corrosion had been assessed using a rabbit skin test (Draize *et al.*, 1944; Draize 1959). However, *in vitro* alternatives have now taken the place of the rabbit test and in a similar manner aim at a basic hazard identification of chemicals which can cause burns or a significant level of acute skin irritation (ECHA, 2008b; Eskes *et al.*, 2012; Commission Regulation, 2009). These efforts in regulatory toxicology are directed towards characterisation of the intrinsic properties of substances, with subsequent application of that knowledge to mixtures and formulations. Hazard information from human studies is unfortunatley not available, since due to ethical reasons, testing in humans for clasification and labelling purposes is not accepted.

In the clinics, reports on acute skin irritation are rare; skin corrosion (chemical burns) do occur, but even so, the actual exposure is often hard to characterise. Utimately nevertheless, the value of any piece of toxicological work is the prediction of effects seen in exposed human population. To obtain controlled human acute skin irritation information, an alternative strategy involving a protocol for the use of human volunteers, the 4 h Human Patch Test (4 h HPT), to characterize skin irritation hazard has been developed and described extensively in the literature (Basketter, 1994; Basketter *et al.* 1994a, b; 1997; York *et al.* 1996; Robinson *et al.*, 2001).

The 4 h HPT provides the opportunity to identify substances with significant skin irritation potential without recourse to the use of animals. It can be applied for the evaluation skin effects of single substances as well as mixtures and formulations (Robinson *et al.*, 2005). The human skin irritation test is very similar to the regulatory accepted *in vivo* rabbit skin irritation test, but it is designed to limit the intensity of skin reactions in human volunteers. The value of the method is in 1) providing data for the identification of those substances or formulation which should or should not be classified as irritant, and 2) providing “gold standard” data for future validations of alternative/*in vitro* methods replacing the *in vivo* rabbit test for classification and labelling purposes in regulatory toxicology.

In the material that follows, the literature has been surveyed to permit the assembly of an extended catalogue of substances to which human subjects have been exposed using the 4 h HPT protocol. Only on very few occasions, substances appeared to possess a greater ability to generate irritant skin reactions than had been expected. More importantly, many more substances had only a very limited effect on skin. Consequently, it is essential that new *in vitro* toxicology tests are calibrated and whenever possible validated against human data rather than use information from *in vivo* rabbit assays obtained usually from outdated databases.

## Material and methods

### The 4 h human patch test – protocol

The human 4 h patch test has been described in complete detail in the literature (Basketter *et al.* 1994a, 1997; York *et al.*, 1996; Robinson *et al.*, 2001, 2005). Briefly, the human patch test procedure involves application of 0.2 ml (0.2g for solid test materials) on a 25 mm plain Hill Top Chamber containing a Webril pad (Hill Top Companies, Cincinnati, Ohio, USA), moistened for solid test materials, to the skin of the upper outer arm of 30 human volunteers for up to 4 hours.

To avoid the production of unacceptably strong reactions, test materials are applied progressively from 15 and 30 minutes through 1, 2, 3 and 4 h. Each progressive application is at a new skin site. The shorter exposure periods can be omitted if the study directors are satisfied that excessive reactions will not occur following longer exposure. Treatment sites are assessed for the presence of irritation at 24, 48 and 72h after patch removal. A volunteer with a reaction at any of the assessments is considered to have demonstrated a “positive” irritant reaction and treatment with the causative substance does not proceed on that person. For panellists with a “+” or greater response at application times of less than 4 h, it is assumed that they would present a stronger irritant reaction if exposed for 4 h. However, once a “+” or greater response is obtained, there is no need to subject these panellists to further treatment with that substance. In evaluating the results, what is measured is the number of panellists who had a positive “irritant” reaction after a 4-h exposure. If irritation reactions to the undiluted test substance is an significantly greater than or not significantly different (using Fisher's exact test) from the level of reaction in that same panel of volunteers to 20% SDS, the substance should be classified as irritant to skin (I); where the level of reaction is substantially and statistically significantly lower than the response to SDS, the substance is not classified (NC) (Basketter *et al.*, 1997). Very occasionally, where the response is significantly stronger (and faster to occur), *e.g.* to 0.5% NaOH, then the substance is suggested to be a potential corrosive (C).

In all the above mentioned studies, 20% sodium dodecyl sulfate (SDS) was used as positive control, for reasons that have been well documented (Basketter *et al.* 1994a; York *et al.*, 1996; Robinson *et al.*, 2001). A minimum of one third of the panel should react to SDS for the study to be regarded as valid, although exception may be made, *e.g.* when a large proportion of the panel react to the test substance.

## Results

The results of the human 4 h patch tests conducted on 81 substances are presented in Table 1, together with their CAS numbers and experimental results. These data have been collated from three main publications (Robinson *et al.*, 2001; Basketter *et al.*, 2004; Jirova *et al.*, 2010). Table 1 also reports the proportion of test subjects reacting to the test substance as well as their response to the concurrent 20% SDS positive control. From this information, the final column records how the materials should be classified on the basis of the human response. It is important to mention that use of the positive control in each experiment has compensated for the inevitable variation that occurs beween different human volunteer panels. Furthermore, it has already been demonstrated that the presence of atopicity, and factors such as gender, ethnicity, age, geography and season have no impact on the conclusions drawn from the results (Griffiths *et al.*, 1996; Basketter *et al.*, 1996a, b; McFadden *et al.*, 1997; Robinson *et al.*, 1998, 1999 2001).

**Table 1:** Materials tested in the human 4 hour patch test.

No. Test substance		CAS No.	Source of data^1^	Known in vivo class	Classification in 4 h HPT^2^	4 h HPT positive^3^	% of positive reactions^4^	SDS positive^5^
1	Acetic acid (10%)	64-19-7	Basketter *et al.*, 2004	R38	NC	6/63	9.5	45/64
	Acetic acid (10%)	64-19-7	Robinson *et al.*, 2001*	R38	NC (I)	15/46	32.6	76/98
2	Alcohol ethoxylate C_11_/E3	*not allocated*	Basketter *et al.*, 2004	R38	NC	1/32	3.1	26/32
3	Alcohol ethoxylate C_11_/E7	*not allocated*	Basketter *et al.*, 2004	R38	NC	0/31	0	12/31
4	Alcohol ethoxylate C_12-15_/E3	*not allocated*	Basketter *et al.*, 2004	R38	NC	0/32	0	24/32
5	Alcohol ethoxylate C_12-15_/E5 phosphate	*not allocated*	Basketter *et al.*, 2004	R34	NC	1/32	3.1	23/33
6	Alcohol ethoxylate C_16-18_/E5	*not allocated*	Basketter *et al.*, 2004	R38	NC	0/27	0	14/27
7	Alcohol ethoxylate C_16-18_/E14	*not allocated*	Basketter *et al.*, 2004	R38	NC	0/27	0	14/27
8	Alkyl dimethyl betaine	68424-94-2	Basketter *et al.*, 2004	R38	NC	3/32	9.3	12/32
9	Alkyl polyglucoside 600	*not allocated*	Basketter *et al.*, 2004	NC	NC	1/30	3.3	28/31
10	Benzalkonium chloride (7.5%)	63449-41-2	Basketter *et al.*, 2004 Robinson *et al.*, 2001 *	R38	I	19/56	33.9	32/56
11	Benzyl alcohol	100-51-6	Basketter *et al.*, 2004	NC	NC	1/31	3.2	17/32
12	Benzyl salicylate	118-58-1	Basketter *et al.*, 2004	NC	NC	0/30	0	20/31
13	Butan-1-ol	35296-72-1	Basketter *et al.*, 2004	NC	NC	1/31	3.2	15/31
14	1-Bromo-4-chlorobutane	6940-78-9	Jirova *et al.*, 2010	NC	NC	0/30	0	22/30
15	1-Bromohexane	111-25-1	Jirova *et al.*, 2010	R38	I	16/30	53.3	22/30
16	Butyl benzoate	136-60-7	Basketter *et al.*, 2004	NC	NC	0/30	0	14/30
17	Butyl methacrylate	97-88-1	Jirova *et al.*, 2010	R38	NC	0/30	0	22/30
18	Citronellol	106-22-9	Basketter *et al.*, 2004	R38	NC	0/30	0	20/31
19	Cocotrimethyl ammonium chloride	61789-18-2	Basketter *et al.*, 2004 Robinson *et al.*, 2001 *	R38	NC (I)	20/89	22.5	50/90
20	Decanoic acid	334-48-5	Basketter *et al.*, 2004 Jirova *et al.*, 2010	R38	I	82/110	74.5	77/109
21	Decanol	112-30-1	Basketter *et al.*, 2004 Jirova *et al.*, 2010 Robinson *et al.*, 2001 *	R38	NC	25/189	13.2	118/189
22	N,N-Dimethyl-N-dodecyl aminobetaine	*not allocated*	Basketter *et al.*, 2004	R38	I	30/32	93.8	27/32
23	3,4-Dimethyl-1H-pyrazole	2820-37-3	Jirova *et al.*, 2010	NC	NC (I)	11/29	37.9	26/29
24	Dimethylsulfoxide	67-68-5	Basketter *et al.*, 2004	NC	I	31/31	100	12/31
25	Dodecanoic acid	143-07-7	Basketter *et al.*, 2004; Jirova *et al.*, 2010	R38	NC	4/90	4.4	65/91
26	Dodecanol	112-53-8	Basketter *et al.*, 2004	NC	NC	0/29	0	16/29
27	Ethylenediamine tetraacetic acid disodium salt	139-33-3	Basketter *et al.*, 2004	NC	NC	0/26	0	21/26
28	Ethanol	64-17-5	Basketter *et al.*, 2004	NC	NC	1/31	3.2	15/31
29	Eugenol	97-53-0	Basketter *et al.*, 2004	R38	NC	4/26	15.3	21/26
30	Geraniol	106-24-1	Basketter *et al.*, 2004	R38	NC	5/28	17.9	23/30
31	Heptanal	111-71-7	Jirova *et al.*, 2010	R38	I	17/29	58.6	23/29
32	Heptanoic acid	111-14-8	Basketter *et al.*, 2004	R34	I	20/31	64.5	20/31
33	Heptyl butyrate	5870-93-9	Basketter *et al.*, 2004 Jirova *et al.*, 2010	NC	NC	0/60	0	40/61
34	Hexadecanoic acid	57-10-3	Basketter *et al.*, 2004	NC	NC	0/29	0	22/31
35	Hexanol	111-27-3	Basketter *et al.*, 2004	R38	NC (I)	8/28	28.6	21/28
	Hexanol	111-27-3	Robinson *et al.*, 2001*	R38	NC	10/59	16.9	48/58
36	Hexyl salicylate	6259-76-3	Basketter *et al.*, 2004 Jirova *et al.*, 2010	R38	NC	0/60	0	38/60
37	Hydrochloric acid (10%)	7647-01-0	Basketter *et al.*, 2004	R38	NC	16/89	18.0	49/91
38	Hydrogenated tallow amine	61788-45-2	Basketter *et al.*, 2004	R38	I	19/19	100	17/19
39	Hydroxycitronellal	89-43-0	Jirova *et al.*, 2010	NC	NC	0/30	0	22/30
40	Isopropanol	67-63-0	Basketter *et al.*, 2004	NC	NC	0/31	0	17/32
41	2-Isopropyl-2-isobutyl-1,3-dimethoxypropane	129228-21-3	Jirova *et al.*, 2010	R38	NC (I)	6/29	20.7	26/29
42	Isopropyl myristate	110-27-0	Basketter *et al.*, 2004	NC	NC	1/30	3.3	18/31
43	Isopropyl palmitate	142-91-6	Basketter *et al.*, 2004	NC	NC	0/29	0	17/29
44	1-(2-Isopropylphenyl)-1-phenylethane (Mixture of isomers)	191044-60-7	Jirova *et al.*, 2010	R38	NC	0/29	0	26/29
45	Lactic acid	50-21-5	Basketter *et al.*, 2004	R38	I/C	21/26	80.8	15/25
46	Linalyl acetate	115-95-7	Basketter *et al.*, 2004/15	R38	NC	1/61	1.6	35/61
47	Methyl caproate	106-70-7	Basketter *et al.*, 2004	NC	NC	0/29	0	17/29
48	Bis[(1-Methylimidazol)-(2- ethyl-hexanoate)], zinc complex	not allocated	Jirova *et al.*, 2010	R38	NC	0/29	0	26/29
49	Methyl laurate	111-82-0	Basketter *et al.*, 2004	R38	NC	0/31	0	15/31
50	Methyl palmitate	112-39-0	Basketter *et al.*, 2004	NC	NC	1/29	3.5	17/29
51	4-Methylthio benzaldehyde	3446-89-7	Jirova *et al.*, 2010	NC	NC	0/30	0	22/30
52	1-Naphthalene acetic acid	86-87-3	Jirova *et al.*, 2010	NC	NC	0/30	0	22/30
53	Nonanoic acid	112-05-0	Jirova *et al.*, 2010	R34/R38	I	19/29	65.5	26/29
54	Octanol	111-87-5	Basketter *et al.*, 2004	R38	NC	5/28	17.9	21/28
	Octanol	111-87-5	Robinson *et al.*, 2001*	R38	NC	9/55	16.4	48/58
55	Octanoic acid	124-07-2	Basketter *et al.*, 2004	R34	I	48/63	76.2	38/62
56	n-Pentanol	71-41-0	Basketter *et al.*, 2004	NC	NC	0/30	0	14/30
57	Polyethylene glycol 400	25322-68-3	Basketter *et al.*, 2004	NC	NC	0/28	0	12/28
58	di-n-Propyl disulfide	629-19-6	Jirova *et al.*, 2010	R38	NC	6/30	20	22/30
59	di-Propylene glycol	25265-71-8	Jirova *et al.*, 2010	NC	NC	0/30	0	22/30
60	Propylene glycol tertiary butyl ether	57018-52-7	Basketter *et al.*, 2004	NC	NC	0/28	0	12/28
61	Potassium soap	8046-74-0	Basketter *et al.*, 2004	NC	NC	0/31	0	9/29
62	C_12-13_ beta-branched primary alcohol sulfate/1-ethoxylate	*not allocated*	Basketter *et al.*, 2004	R38	NC (I)	9/30	30	28/31
63	C_12-13_ beta-branched primary alcohol sulfate	*not allocated*	Basketter *et al.*, 2004	R38	I	26/31	83.9	28/31
64	Propylene glycol	57-55-6	Basketter *et al.*, 2004	NC	NC	2/32	6.25	23/33
65	Sodium carbonate	497-19-8	Basketter *et al.*, 2004	NC	NC	0/26	0	21/26
66	Sodium dodecyl sulphate (20%)	151-21-3	Basketter *et al.*, 2004	R38	I	54/65	83.1	137/182
	Sodium dodecyl sulphate (20%)	151-21-3	Jirova *et al.*, 2010	R38	I	94/118	79.7	94/118
	Sodium dodecyl sulphate (20%)	151-21-3	Robinson *et al.*, 2001*	R38	I	817/1154	70.8	1154/1154
	Sodium dodecyl sulphate (10%)	151-21-3	Robinson *et al.*, 2001*	R38	NC (I)	203/295	68.8	239/292
	Sodium dodecyl sulphate (1%)	151-21-3	Robinson *et al.*, 2001*	R38	NC (I)	52/231	22.5	196/229
67	Sodium hydroxide (0.5%)	1310-73-2	Basketter *et al.*, 2004	R38	I/C	20/33^5^	60.6^5^	23/33^5^
	Sodium hydroxide (0.5%)	1310-73-2	Robinson *et al.*, 2001*	R38	I/C	57/98^5^	58.2	12/98^5^
68	Sodium percarbonate	15630-89-4	Basketter *et al.*, 2004	R38	NC	1/26	3.8	21/26
69	Sodium perborate	7632-04-4	Basketter *et al.*, 2004	R38	NC	1/26	3.9	21/26
70	Sodium soap	*not allocated*	Basketter *et al.*, 2004	NC	NC	0/31	0	9/29
71	Sodium xylene sulfonate	1300-72-7	Basketter *et al.*, 2004	NC	NC	0/30	0	16/30
72	1-(Spiro[4.5]dec-7-en-7-yl)pent-4-en-1-one (mixture of isomers)	224031-70-3	Jirova *et al.*, 2010	NC	NC	0/29	0	26/29
73	a-Terpineol	98-55-5	Basketter *et al.*, 2004/15	R38	NC	0/59	0	39/59
74	Terpinyl acetate	80-26-2	Jirova *et al.*, 2010	R38	NC	0/30	0	22/30
75	Tetradecanoic acid	544-63-8	Basketter *et al.*, 2004	NC	NC	0/29	0	22/31
76	Tetradecanol	112-72-1	Basketter *et al.*, 2004	NC	NC	0/29	0	16/29
77	Triethanolamine	102-71-6	Basketter *et al.*, 2004	NC	NC	0/32	0	26/32
78	Tris(hydroxymethyl)aminomethane	77-86-1	Basketter *et al.*, 2004	NC	NC	2/32	6.2	12/32
79	Tween 80	9005-65-6	Basketter *et al.*, 2004	NC	NC	1/29	3.5	24/29
	Tween 80	9005-65-6	Robinson *et al.*, 2001*	NC	NC	2/53	3.8	32/56
80	10-Undecenoic acid	112-38-9	Jirova *et al.*, 2010	R38	NC	1/29	3.5	23/29
81	Water	7732-18-5	Basketter *et al.*, 2004	NC	NC	3/59	5.8	58/59

1 Reference source for the original data and classifications

2 Classification of a substance in 4 h HPT based on established prediction model

3 Number of individuals with a positive irritant reaction to the test material/total panel size

4 Percentage of positive responses to substance independently of SDS

5 Number of individuals with a positive irritant reaction to the 20% SDS control in the same panel

6 Number of positive reactions after exposure only up to 1h

* Results from multi-laboratory study (two and more laboratories)

R34-Corrosive, R38-Irritant, NC- not classified; I- Irritant in human, I/C – Irritant possibly corrosive

NC (I) - possible introduction of a provision that where >20% of the panel are positive to the test substance independently of the response to SDS, the substance may be considered as irritant.

Based on *in vivo* rabbit tests, more than 50% of chemicals are classified as irritants in Table 1 (Robinson *et al.*, 2001; Basketter *et al.*, 2004; Jirova *et al.*, 2010), wheras in the human patch test, using the classification critiera described earlier, only about 20% of the substances tested were identified as human irritants, with two possible corrosive classifications (#45 Lactic Acid; #67 0.5% Sodium Hydroxide).

In the current study, 7 substances, namely: #1 Acetic acid, #19 Cocotrimethyl ammonium chloride, #23 3,4-Dimethyl-1H-pyrazole, #35 hexanol, #41 2- Isopropyl-2-isobutyl-1,3-dimethoxypropane. #43 C_12-13__beta-branched_ primary alcohol sulfate/1-ethoxylate, #66 Sodium dodecyl sulphate at concentrations of 10% and 1%, provided significant irritating response in more than 20% of panelists, as is indicated in Table 1. Additional precautionary principle, that positive classification would be assigned if more than 20% of the panel reacted on the test substance, could be applied in this situation to avoid false negative results.

## Discussion

### Regulatory relevance of human data

According to the European CLP Regulation (Commission Regulation, 2009), classification of any substance or mixture should preferably be generated in accordance with the test methods referred in Regulation (EC) No. 1907/2006, *i.e.* Council Regulation (EC) No. 440/2008 or OECD Guidelines. However, the CLP Regulation at the same time stipulates in the Recital 20–21 and Article 7.3, that data obtained from other sources, such as clinical studies, can be used for the purpose of the CLP Regulation (ECHA, 2008a). Classification should be carried out on the basis of all relevant information on the hazards of the substance or mixture and there is an obligation to evaluate the quality of all available information.

It is important to keep in mind that the classification of a substance as irritant in existing *in vivo* protocols used for regulatory toxicology purposes reflects only a significant potential of substance for the production of an acute irritant effect. The cumulative irritant capability of a substance is not taken into account. Regulatory decision not to classify a substance, mixture or formulation does not by any means imply that the product is entirely free of any skin irritation potential, only that the level of irritant activity is likely not sufficient to trigger classification.

Although it is not allowed to test substances on humans for the purpose of CLP Regulation, the manufacturer, importer or downstream user should, for the purpose of classification, take into account all human data available, such as epidemiological studies on exposed populations, accidental or occupational exposure data, and clinical studies. That information should be compared with the criteria for the different hazard classes and differentiations, so that the manufacturer, importer or downstream user can arrive at a conclusion as to whether or not the substance or mixture should be classified as hazardous.

Reflecting on results presented in Table 1, the current classification decision strategy based on human 4 h patch test states that substance whose irritant capacity is significantly less than 20% SDS should not be classified. However, this conclusion might require some reconsideration. Under conditions, where a panel of volunteers is large and the statistical significance of Fisher′s exact test and final classification may be influenced, the provision could be included, that positive classification would normally occure if more than 20% of panellist reacted to the test substance, also considering the precautional principle for later accidental exposure in humans. In this case, a recommended number of panellists involved in the study should be defined.

The quality and relevance of existing human data for hazard assessment should always be critically reviewed. There may be a significant level of uncertainty in existing human data due to poor reporting and lack of specific information on exposure. Diagnosis confirmed by expert physicians may be missing. Confounding factors may not have been accounted for. Small group sizes may flaw the statistical strength of evidence and many other factors may compromise the validity of human data. In clinical and scientific studies the selection of individuals for the test and the control groups must be carefully considered. Any clinical studies may however contribute to the weight of evidence assessment with other available information such as existing data from animal or other experimental studies.

Importantly, when human data demonstrate hazards that have not been identified by animal studies, the animal results should be weighed against human data and expert judgement should be used to ensure the best protection of human health when evaluating both the animal and human data, as specified in Recital 28 of the CLP Regulation. Actually, the available data indicate that human skin is, in most cases, less sensitive than that of rabbits (Phillips *et al.*, 1972; Nixon *et al.*, 1975; Campbell & Bruce, 1981).

A critical review of the value of human studies is provided in IR/CSA Section R.4.3.3 and more specific considerations for the skin corrosion/irritation endpoint are given in IR/CSA Section R.7.2.4.2. IR/CSA Guidance on Information Requirements and Chemical Safety Assessment, ECHA, 2008 (http://guidance.echa.europa.eu/docs/guidance_document/information_requirements_en.htm).

### Use of human data for development of relevant *in vitro* assays

*In vitro* alternatives for the identification of skin irritation have been the subject of investigation and development in a considerable number of laboratories for many years (reviewed in Eskes *et al.*, 2012; Welss *et al.*, 2004; Gibbs, 2009), and these are now broadly accepted by regulatory authorities (ECHA, 2008b; Commission Regulation, 2009). These alternatives were established to recapitulate the results previously obtained from *in vivo* rabbit studies, which are very sensitive, however they poorly reflect human exposure scenarios and thus also human hazard (Phillips *et al.*, 1972; Nixon *et al.*, 1975; Campbell & Bruce, 1981).

Clinically, skin irritation is a type of dermatitis whose causation is complex and which involves repeated exposures to a range of noxious stimuli. Skin corrosion, where substances can cause burns and irreverisble damage is a much more clear cut situation. Because of the intensity of the skin responses to corrosive substances and the irreversibility of effects, correct prediction of corrosive effect is of great importance. Thus, incorrect classification of corrosive substances, either by the *in vivo* rabbit assay or by *in vitro* methods established on the rabbit based classification, remains a cause of some concern.

The data presented in Table 1 offer results with 81 substances which can be used to assess the ability of *in vitro* methods to predict accurately the acute skin irritation and corrosion potential of a range of substances. The results include two substances, lactic acid and 0.5% sodium hydroxide, which based on rabbit data were not thought to be potentially corrosive, but for which the results of the human study suggest corrosive classification. Correct classification of lactic acid and NaOH including their dilutions is of specific importance as they are used as ingredients in consumer products (cosmetics) for keratolytic purposes. It is of concern whether keratolysis based on skin corrosive effect for cosmetic purposes should be generally acceptable.

## Conclusion

The retrospective evaluation of existing human data presented in this paper provides n unique opportunity to compare data on skin irritation hazard classification obtained with classic regulatory acccepted methods (*i.e.* the *in vivo* rabbit skin irritation test) with human data on hazard, with the ultimate aim to enhance the accuracy of the information on hazard contained in manufacturers’ safety data sheets. The information presented in Table 1 can and should be used to develop alternative methods that provide classification and labelling that is most relevant to the true human hazard.
